# Does clinical exposure to different skin tones during training improve diagnostic ability?

**DOI:** 10.1111/medu.70088

**Published:** 2025-12-12

**Authors:** Yusra Shammoon, Anna Coulson, Bethan Trigg, Nariell Morrison, Adam Potts, Moira Pain, Olamide Oguntimehin, Eleanor J. Hothersall‐Davies, Richard Hankins, Celia A. Brown, Amir H. Sam

**Affiliations:** ^1^ Imperial College School of Medicine Imperial College London London UK; ^2^ General Medical Council Manchester UK; ^3^ University of Dundee Dundee UK

## Abstract

**Background:**

Previous studies have shown that medical students demonstrate poorer performance when diagnosing pathology in skin of colour (SOC) compared to white skin (WS); it is important to understand the reasons underpinning this. If not addressed, poorer differential diagnostic ability in certain skin tones could entrench existing racial inequities in health care. We investigated whether exposure to a predominant patient skin colour during clinical practice affects diagnostic ability in WS and SOC.

**Methods:**

Participants were international medical graduates (IMGs) and medical students from Imperial College London and the University of Dundee, recruited between January and May 2024. Participants were divided into two groups, based on whether they were predominantly exposed to white patients (WP) or non‐white patients (NWP) in their practice. Participants sat a dermatology quiz, in which they were asked to provide a diagnosis for 22 image‐based vignettes, covering 11 clinical presentations, each shown in WS and SOC. For each of the WP and NWP exposed groups, we compared their diagnostic ability in WS and SOC presentations.

**Results:**

A total of 411 participants were analysed; 187 predominantly exposed to WP and 224 predominantly exposed to NWP. Both groups demonstrated a statistically significantly better diagnostic ability in WS compared to SOC (p < 0.01). Overall, there was no significant difference in differential diagnostic ability in WS and SOC between the WP‐exposed and NWP‐exposed groups (p = 0.731).

**Discussion:**

Regardless of the predominant patient skin colour participants saw in their practice, participants were worse at diagnosing pathology in SOC. This highlights that clinical exposure to SOC is not sufficient to mitigate clinicians' inferior diagnostic ability in non‐white skin tones. Therefore, effort must be made to improve the diversity of skin colours represented in medical education resources, to improve clinicians' familiarity with pathology in different skin tones and minimize the risk of patients being misdiagnosed due to their skin colour.

## Abstract 2

**Clinical exposure isn't enough.** Medical students and IMGs diagnose skin of colour less accurately, highlighting the need for better representation in education.

## 1 BACKGROUND

Racial inequity exists within health care.^1^, ^2^ This manifests in several ways, including unequal access to care, discrepancies in care quality and reduced trust and engagement with health care services.^2^ During the COVID–19 pandemic, reports published by Public Health England highlighted racial disparities in health outcomes, with individuals from black and minority ethnic groups experiencing disproportionate adverse effects on morbidity and mortality.^3^

Studies have suggested that clinicians may have poorer diagnostic ability in skin of colour (SOC): a US survey of dermatology residents found that doctors were less likely to biopsy malignant conditions in those with darker skin, compared to lighter skin (odds ratio 0.42),^4^ whereas UK clinicians were more likely to report ‘unknown’ dermatological diagnosis for Indian, Bangladeshi and Pakistani (4.8%) and African/Afro‐Caribbean (3.3%) patients compared to White British/Irish patients (0.09%).^5^ Moreover, a scoping review has found that pathologies presenting in darker skin tones are at risk of being mismanaged due to health professionals holding a relative lack of confidence in their treatment.^6^ Poorer diagnostic ability in SOC can emerge as early as medical training. A study at a UK medical school showed that students were more confident and able to diagnose dermatological conditions in white skin (WS) compared to non‐white skin when answering image‐based clinical questions.^7^ These findings are concerning, as reduced diagnostic ability and confidence may contribute to misdiagnosis and inappropriate treatment for patients, propagating further health inequalities.

Racial inequity in health care is multifactorial— there is evidence it stems partly from our education systems.^8^ A review of clinical images in popular medical textbooks identified that, although images reflected the national race distribution in the USA, lighter skin tones were over‐represented.^9^ Similar under‐representation of SOC has been highlighted in other resources, including preparatory materials for the United States Medical Licensing Examination (USMLE) and medical school lectures.^10^, ^11^, ^12^, ^13^

Medical educational resources and clinical exposure are mainstays in familiarizing doctors with clinical presentations and developing diagnostic competence. Clinical exposure includes the patients that students and doctors encounter throughout training and consultations, during clinical attachments. In light of the existing gaps in medical education resources, we therefore aimed to investigate whether the ethnicity of the patient population that medical students and clinicians are exposed to impacts their diagnostic ability in WS and SOC. We hypothesized that if students and clinicians are exposed to a predominant patient skin colour in their training, it may influence their comparative diagnostic ability.

Identifying gaps in diagnostic ability between WS and SOC, and the possible causative factors for these, is crucial as this would enable targeted educational interventions to address knowledge deficits and help to reduce health inequities.

## 2 METHODS

### 2.1 Study design

We conducted a multi‐site, cross‐sectional study. Ethical approval was obtained from the Imperial College London Education Ethics Review Panel (EERP2324‐043a).

### 2.2 Study participants and recruitment

We recruited three groups of study participants: final year medical students at the University of Dundee, final year medical students at Imperial College School of Medicine (ICSM) and international medical graduates (IMGs) who were sitting the General Medical Council Professional and Linguistics Assessments Board 1 (PLAB1) exam.

Our participant groups were chosen as they are exposed to patient populations of varying ethnicities. For Dundee students, their patient population is predominantly white (89.9% as per 2022 census data).^14^ For Imperial students, their training area represents the most ethnically diverse patient population in the UK (46.2% of the local population identify with Asian, Black, mixed or ‘other’ ethnic groups).^15^ For IMGs sitting the PLAB1, over 70% are from countries with non‐white majority populations, based on internal General Medical Council (GMC) data.

Participants were recruited between January and May 2024. All final year medical students at the University of Dundee and ICSM were invited to take part in the study quiz, advertised and delivered during whole year group lectures. IMGs registered for the February and May PLAB1 sittings were invited to participate in the quiz via email correspondence disseminated by the GMC.

Participants were not offered a financial incentive for participating in the study, and participation was voluntary. No identifying information was collected and, as such, all responses were anonymous.

### 2.3 Quiz design

We used a pre‐existing dermatology quiz that had been designed by ICSM for use in a previous study^7^. The quiz comprised 22 distinct cases based on 11 different clinical presentations, but had not itself been validated. Each case image was taken from a different individual patient. Each case included a clinical vignette and a corresponding image. Presentations included cellulitis, central cyanosis, chickenpox, eczema, Henoch‐Schönlein purpura (HSP), Kawasaki disease, Lyme disease, meningococcal rash, pityriasis versicolor, shingles and urticaria. For each presentation, image‐based vignettes were provided in both WS and SOC. SOC images included a range of skin tones, including Black and Asian ethnic groups. To minimize practice effects, the vignette stems for each skin colour within a given presentation were varied slightly rather than presented identically. Questions were ordered so that WS and SOC vignettes for the same presentation were not consecutive. All participants saw the 22 questions in the same order.

Each participant was asked for consent for their data to be used in research at the start of the quiz; individuals could still complete the quiz even if they opted out from having their data used. Prior to commencing the questions, participants were asked to indicate the majority ethnicity of the patient population to which they were most exposed. Each participant was then asked to state a diagnosis for each clinical case vignette, with their answers entered as free text – no explanation or clinical reasoning for their answers was required. Participants were given two minutes per vignette to complete their response. Participants worked individually, although we cannot guarantee they did not access online resources.

### 2.4 Data collection

The quiz session was delivered through an in‐person lecture for the University of Dundee and online for the ICSM groups. For IMGs, the quiz session was delivered multiple times, through an online webinar and at parallel in‐person sessions at four sites (Birmingham, Edinburgh, Oxford and London) following the PLAB1 exam. The multi‐session approach was required for IMGs to accommodate varying time zones and work patterns, since our IMG participants came from across the globe.

Regardless of the mode of session facilitation, all participants accessed the quiz via a cloud‐based tool (Mentimeter; https://www.mentimeter.com). Participants were asked to scan a QR code via their smart device to complete the quiz. For each presentation, participants were asked to type in the relevant diagnosis. All quiz facilitators received comprehensive briefings to ensure quiz delivery was standardized.

### 2.5 Data analysis

Answers submitted by study participants were exported from Mentimeter and collated in Microsoft Excel. Responses from participants who did not consent for their data to be used were removed from the dataset. Responses from participants who did not substantially complete the quiz (leaving before progressing through 80% of the quiz) were also removed. Quiz answers were marked by a group of four researchers. A predetermined list of correct answers was generated by the group of researchers. Where answers did not match the predetermined list, researchers would collectively discuss and decide whether to accept alternative answers. Where alternative answers or spelling mistakes were accepted, these variations were consistently allowed for all participants.

Participants were then split into two groups for data analysis, based on their stated majority patient ethnicity they were exposed to: WP (majority patients of white ethnicity) and NWP (majority patients of non‐white ethnicity). Participants in the WP group were those who reported their majority patient ethnicity as ‘White’. Participants in the NWP group were those who reported their majority patient ethnicity as: ‘Asian or Asian British’; ‘Black, Black British, African, or Caribbean’; ‘Mixed or Multiple ethnic groups’; and ‘Other ethnic group’. Participants who declined to state their patient population ethnicity were not included in the analysis.

Data management and analysis were conducted using Microsoft Excel and SPSS v.26. For each participant, a total paired difference (TPD) score for diagnostic ability was calculated based on their marks in WS and SOC cases. This was the sum of the paired difference score for each condition for each participant. Table 1 shows how the paired difference score for each condition was calculated, giving a possible range in TPD score of −11 to +11. Positive scores indicate a better diagnostic ability in WS than in SOC, whereas negative scores indicate a better diagnostic ability in SOC than WS. Given the known poorer diagnostic ability for SOC patients, we chose to calculate differences to reflect that a positive score would show an exacerbation of the existing trends and suggest worse health outcomes for patients with SOC.

**TABLE 1:** Paired difference score allocated for each condition (determined by scores in both the white skin (WS) and skin of colour (SOC) vignettes) for each participant.

Paired difference score allocated		
‐1	0	+1
• WS vignette incorrect and SOC vignette correct	Both WS and SOC vignettes correct Both WS and SOC vignettes incorrect	WS vignette correct and SOC vignette incorrect

TPD scores in both the WP and NWP groups were approximately normally distributed, and thus parametric methods were used for analysis. For each of the WP and NWP groups, the mean TPD score was calculated. A one‐sample t‐test (critical p‐value of 0.025) was used to test the null hypothesis that the mean TPD for each group was zero, which would indicate that there was no difference in diagnostic ability between WS and SOC. A two‐sample t‐test (critical p‐value of 0.05) was used to test the null hypothesis that there was no difference in mean TPD (i.e. the differential diagnostic ability in WS and SOC) between the WP and NWP groups.

To elucidate our findings further, we examined the responses of participants who, for each condition, gave the correct diagnosis for one patient's skin colour and the incorrect diagnosis for the other. Within each patient population group, the proportion of these participants who gave the correct diagnosis for the patient with WS was calculated and compared to the ‘equal ignorance’ level of 0.5 by calculating the 99.8% confidence interval for the proportion (which adjusts for multiple comparisons). The difference in the proportions between the WP and NWP groups was examined using a two‐sided test of proportions with a critical p‐value of 0.0045.

### 2.6 Statistical power

The primary outcome for this study was the difference in mean TPD between the WP and NWP participant groups. A two‐sided sample size calculation was undertaken in GPower 3.1.9.7, with an alpha of 0.05 and 80% power. The study sought to detect a small‐to‐medium Cohen's d effect size of 0.3 standard deviations between groups, which required 176 participants in each population group.

## 3 RESULTS

### 3.1 Participants

In total 416 participants completed the quiz. Five participants were excluded, as they declined to state their predominant patient ethnicity. A total of 411 participants were included in the analysis. Baseline characteristics for participants can be seen in Table 2.

**TABLE 2:** Distribution of participants by recruitment group and the predominant patient ethnicity they had been exposed to.

Recruitment group	WP	NWP			
	White	Asian or Asian British	Mixed or multiple ethnic groups	Black, Black British African or Caribbean	Other ethnic group
Imperial	76 (40.6)	33 (14.7)	68 (30.4)	1 (0.4)	0 (0.0)
Dundee	94 (50.3)	1 (0.4)	5 (2.2)	0 (0.0)	0 (0.0)
IMGs	17 (9.1)	40 (17.9)	28 (12.5)	40 (17.9)	8 (3.6)
Total	187 (100.0)	74 (33.0)	101 (45.1)	41 (18.3)	8 (3.6)
**Total by Analysis Group**	**187**	**224**			

Values are presented as n (%), where percentages indicate the proportion of participants with the specified characteristics within the respective white patient (WP) and non‐white patient (NWP) groups.

There were 187 participants in the WP group and 224 participants in the NWP group. The WP group was predominantly made up of participants from the University of Dundee and ICSM. The NWP group was predominantly composed of IMG and ICSM participants.

### 3.2 Paired difference scores – diagnostic ability in WS and SOC between WP and NWP groups

Both groups were significantly better at diagnosing conditions in WS compared to SOC, with mean TPDs greater than 0 (WP mean TPD = 0.40, SD = 1.66, t(186) = 3.29, *p* = 0.001; NWP mean TPD = 0.46, SD = 1.77, t(223) = 3.89 *p* < 0.0001). A small Cohen's d effect size was demonstrated for both groups (WP = 0.24, NWP = 0.26).

There was no significant difference in TPD between the WP and NWP groups (difference in mean TPD = −0.06, SE of difference = 0.17, t[409] = 0.34, *p* = 0.731). This indicates that both WP and NWP groups exhibited a comparable difference in diagnostic ability between WS and SOC.

### 3.3 Differences in diagnostic ability by condition, patient skin colour and predominant patient population

**FIGURE 1:**
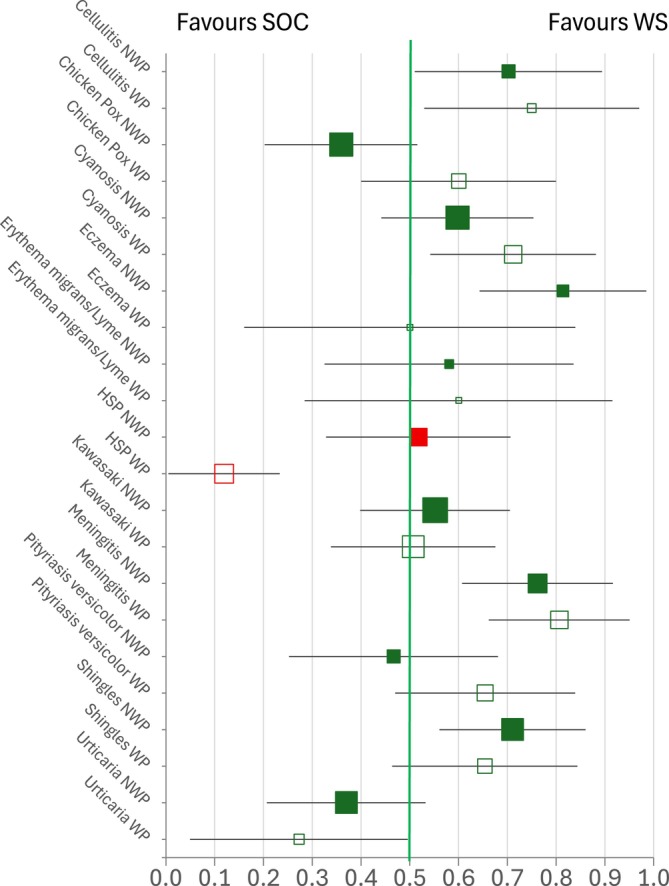
Forest plot showing the proportion of participants with a ‘correct answer bias’ towards white skin (WS) versus skin of colour (SOC) across conditions, separated by candidates predominantly exposed to non‐white patients (NWP) and white patients (WP). Box size is proportional to the number of participants providing one correct and one incorrect answer. The green vertical line at 0.5 indicates equal likelihood of correct responses for WS and SOC. Red markers denote statistically significant differences between NWP and WP groups (critical *p* < 0.0045). Error bars show 99.8% confidence intervals. [Color figure can be viewed at wileyonlinelibrary.com]

Figure 1 shows, for each condition, the proportion of participants with a correct answer for one patient skin colour and an incorrect answer for the other who were correct on WS and incorrect on NWS. The number of participants in these analyses ranged from 18 to 87. Of the 22 comparisons on the graph, 16 showed a trend towards better diagnosis on WS compared with SOC, with seven of these being statistically significant at *p* < 0.002 (the confidence intervals do not cross the vertical green line). Five comparisons showed a trend towards better diagnosis on SOC, with one of these being statistically significant. The final comparison was a proportion of exactly 0.5, i.e. equal diagnostic ability across WS and SOC. The proportions getting one skin colour correct and one incorrect were different across predominant patient populations for only one condition, HSP. Here, participants in the WP group were just over four times more likely to correctly diagnose HSP in SOC, whereas those in the NWP group showed no clear difference (p < 0.001). Full results are shown in Table 3.

**TABLE 3:** Performance of participants predominantly exposed to non‐white patients (NWP) and white patients (WP) across conditions presented in white skin (WS) and skin of colour (SOC).

CONDITION	Exposed to non‐white patients (NWP)				Exposed to white patients (WP)				Difference in percentages (WP‐NWP)	p‐value (critical p = 0.0045)
	% correct WS	% correct SOC	N with 1 correct and 1 incorrect (out of 224 participants)	Of those with 1 correct and 1 incorrect, % with WS correct	% correct WS	% correct SOC	N with 1 correct and 1 incorrect (out of 187 participants)	Of those with 1 correct and 1 incorrect, % with WS correct		
**Cellulitis**	90.2	81.7	47	70.21	93.0	84.5	32	75.00	4.79	0.357
**Chicken Pox**	64.7	74.6	78	35.90	78.1	72.7	50	60.00	24.10	0.009
**Cyanosis**	67.0	59.8	82	59.76	81.3	67.9	59	71.19	11.43	0.144
**Eczema**	92.0	79.9	43	81.40	91.4	91.4	18	50.00	−31.40	0.024
**Erythema migrans/Lyme**	82.6	80.4	31	58.06	85.0	82.9	20	60.00	1.94	0.395
**HSP**	38.4	37.5	58	51.72	33.7	61.0	67	11.94	−39.78	**<0.001**
**Kawasaki**	38.4	34.4	87	55.17	34.2	33.7	73	50.68	−4.49	0.340
**Meningitis**	78.1	63.4	63	76.19	88.8	68.4	62	80.65	4.45	0.332
**Pityriasis versicolor**	37.5	38.8	45	46.67	42.2	33.2	55	65.45	18.79	0.064
**Shingles**	65.6	51.3	76	71.05	81.3	72.7	52	65.38	−5.67	0.318
**Urticaria**	63.8	72.3	73	36.99	74.3	82.4	33	27.27	−9.71	0.239

## 4 DISCUSSION

To our knowledge, this is the first study examining whether clinical exposure to different patient skin tones during training influences diagnostic ability in WS and SOC. Consistent with previous literature, we found that both medical students and doctors are worse at diagnosing pathology in SOC compared to WS. In addition, we demonstrate that the better diagnostic ability in WS compared to SOC was seen regardless of the majority patient ethnicity that participants were exposed to.

It should be noted that while we did observe differences in diagnostic ability in WS and SOC presentations, the effect size for total scores by skin colour was small. This reflects that variation in diagnostic accuracy was at least partly condition‐specific (where large positive and negative paired difference scores for individual conditions partly cancelled each other out when calculating total scores across the quiz). Nevertheless, combining the results for the two participant groups, we find that, on average, there would be one additional diagnostic error in SOC patients for every 25 pairs of WS/SOC patients. Single centre studies of medical students have identified students as poorer at diagnosing squamous cell carcinoma, atopic dermatitis, urticaria and psoriasis in patients with SOC.^16^, ^17^ It would be interesting to conduct further studies, to delineate whether there are specific presentations for which there are larger differences in diagnostic ability depending on patient skin tone and whether this is affected by the patient ethnicity clinicians are exposed to, as well as to explore why diagnostic error occurs using theories of clinical reasoning.^18^

## 5 LIMITATIONS

Our study has several limitations. Although we split our analysis groups based on their exposure to patients of different ethnicities, in reality ‘non‐white’ patients have a range of skin tones, from light to darker shades. We also made the assumption that patients with “Mixed or Multiple ethnic group” backgrounds were “non‐white”, but again, this may not reflect the diversity of skin tones that patients encompass. Therefore, ethnicity alone is an imperfect proxy for estimating the skin tones that health care professionals have observed in their training. However, it was felt to be the best practical study measure, since estimating patient skin tone would be highly subjective between participants.

Regarding our quiz, it is challenging to identify the extent to which participants relied on the text in the vignette rather than the clinical image when determining a diagnosis. Although we could not make the vignettes for the same presentations in WS and SOC completely identical, which may have influenced participants' responses, we ensured they were as similar as possible. We did not want to ask quiz questions without a clinical vignette, as we felt this would not be representative of real‐life clinical reasoning. Similarly, the quality of the photos varied slightly across cases, which may have influenced the ability of participants to make an accurate diagnosis. Further work to validate the quiz against other measures of diagnostic ability would be useful.

There was significant heterogeneity within our participant groups, including variation in individual participants' educational placements, internal medicine teaching and personal characteristics (e.g. ethnicity) that could have affected performance. Furthermore, where participants grew up and where they trained were potential confounds that could not be examined. Our WP group predominantly consisted of medical students, compared to our NWP group; it can be assumed that IMG participants (as qualified clinicians) would have more clinical experience than student participants. Nevertheless, despite differences in training stage, there was no significant difference in our analysis groups' relative performances in WS and SOC. We accepted participant heterogeneity as a limitation of our study, as it is challenging to completely control for a standardized educational experience whilst maintaining recruitment numbers. However, future research could formally investigate the impact of demographic factors and training experience, as well as explore other reasons for differences in diagnostic ability and potential ways to reduce them. We were unable to calculate response rates from all potential IMG participants as denominators were unknown; therefore, we could not assess the representativeness of our study sample.

It should be noted that the diagnostic process may vary when assessing WS and SOC. Anecdotally, some of our IMG participants reported using rash palpation or vital signs to inform the diagnosis of pathology in darker skin tones in real life. It could be that our vignettes did not provide all the cues that participants would normally use to assess clinical presentations in SOC, which could also account for the relatively poorer performance in SOC.

Regardless, our study suggests that clinical exposure to SOC is not sufficient to remove the diagnostic gap between WS and SOC. We must therefore improve training around clinical presentations in SOC, including promoting the inclusion of a diverse range of skin tones in medical education resources. There have been studies reporting initiatives aimed at promoting familiarity with clinical presentations in darker skin tones, including targeted lectures and learning modules, which have improved students' performance in identifying pathology in darker skin.^19^, ^20^ Artificial intelligence is also being explored to broaden repositories of clinical images, to better reflect typically under‐represented skin tones.^21^

## 6 CONCLUSION

Medical education institutions, at both undergraduate and postgraduate levels, should consider adopting initiatives to embed teaching and assessment on clinical presentations across diverse skin tones. Failure to do so risks further entrenching existing racial inequities in health care. This is particularly important given that populations in the UK and USA are set to become increasingly ethnically diverse, with white skin projected to become a “majority‐minority” in the USA by 2043.^22^, ^23^ Thus, clinicians must be equipped with the knowledge and skills required to meet the needs of their evolving patient populations.

## AUTHOR CONTRIBUTIONS

Yusra Shammoon, Anna Coulson, Bethan Trigg, Nariell Morrison, Adam Potts, Moira Pain, Olamide Oguntimehin, Eleanor J. Hothersall‐Davies, Richard Hankins, Celia A. Brown and Amir H. Sam contributed to the conception, data collection, interpretation, and drafting of the work. All authors reviewed and approved the manuscript.

## ETHICS STATEMENT

Ethical approval was obtained from the Imperial College London Education Ethics Review Panel (EERP2324‐043a).

## Data Availability

No data was obtained from outside sources for this study.
